# Significance of Homocysteine Levels in the Management of Polycystic Ovarian Syndrome: A Literature Review

**DOI:** 10.7759/cureus.11110

**Published:** 2020-10-23

**Authors:** Varshitha Kondapaneni, Sai Dheeraj Gutlapalli, Sujan Poudel, Mehwish Zeb, Ijeoma A Toulassi, Ivan Cancarevic

**Affiliations:** 1 Internal Medicine, California Institute of Behavioral Neurosciences & Psychology, Fairfield, USA

**Keywords:** pcos, homocysteine levels

## Abstract

Polycystic ovarian syndrome (PCOS) is a complex disorder involving cardiovascular, metabolic, endocrine, and reproductive systems. Even though the exact etiology is not clear, many studies suggest genetic and environmental factors play a role. Homocysteine (Hcy) is considered to be an independent risk factor for atherogenic and thrombotic components of various systems. Many studies in the past have evaluated Hcy levels in the PCOS population. This article aims to elaborate on the importance of Hcy levels in the overall management of PCOS. We conducted a PubMed data search using combined keywords PCOS and homocysteine levels and manually screened relevant articles for the review while avoiding duplication of data. After the literature review, we analyzed the relationship between homocysteine levels and various components of PCOS. Most of the studies identified a statistically significant elevation in Hcy levels in PCOS women with insulin resistance, androgen excess, elevated markers of cardiovascular risk, recurrent pregnancy loss, and metformin treatment. We also examined studies that focused on treating hyperhomocysteinemia (Hhcy) in PCOS women. However, because of the limited sample sizes and various inclusion criteria used for subjects in the studies, their clinical implication is unclear in routine practice. Furthermore, we encourage clinicians to follow up on Hcy levels in PCOS women at high risk for any complications in their management course. We believe an observational study on a larger scale in a well-defined PCOS population would be useful to uncover the prevalence of elevated Hcy levels in PCOS women, which would help pave the way for establishing treatment guidelines on serum Hcy levels in PCOS management.

## Introduction and background

Polycystic ovarian syndrome (PCOS), also called Stein Leventhal Syndrome, is a complex, multifaceted disorder, with a global prevalence that varies from 2.2% to as high as 26% [1,2]. As of 2010, World Health Organization estimates show that it affected 116 million women worldwide (3.4% of the population) [2]. Among women of reproductive age, PCOS is the most common endocrine disorder affecting metabolic and cardiovascular health apart from reproductive health [3,4].

According to the 2003 revised Rotterdam criteria, PCOS is diagnosed when two out of the three following conditions are present after excluding other possible conditions that mimic PCOS phenotype: (1) clinical and/or biochemical signs of hyperandrogenism, (2) oligo‐ and/or anovulation, and (3) polycystic ovaries on ultrasound examination [5]. Although the exact etiology of PCOS is unknown, many studies strongly suggest genetic and environmental factors play a role in its causation [1,4]. Interestingly, recent studies identified a male PCOS equivalent syndrome in males related to women with PCOS. These males present with similar metabolic, clinical, and hormonal alterations as in PCOS women, which emphasizes a strong genetic component involved in the etiology of PCOS apart from lifestyle, ethnic origin, race, and other environmental factors [4,6]. PCOS and metabolic syndrome share common metabolic and cardiovascular complications, as insulin resistance is the potential pathogenetic mechanism for both [4]. Various metabolic, cardiovascular, clinical components of PCOS include obesity, impaired glucose tolerance, type 2 diabetes, hypertension, coronary vascular disease, obstructive sleep apnea, association with cancer, and dermatological manifestations of hyperandrogenemia [4].

Previous studies have demonstrated that compared with controls, serum homocysteine levels are significantly associated with insulin resistance and hyperinsulinemia in women with PCOS [7,8]. Homocysteine (Hcy) is a sulfur-containing amino acid present at the intersection of two pathways, remethylation and trans-sulphuration, which forms methionine and cysteine, respectively [3,9]. This amino acid metabolism requires vitamin B12, folate, and vitamin B6 as cofactors at different steps of the pathways [9]. Hyperhomocysteinemia (Hhcy) is documented to be an independent risk factor for atherosclerotic vascular disease and thromboembolic disorders [3,9]. Any defect in the enzymes involved in these pathways, deficiencies of vitamin cofactors, hyperinsulinemia, or drugs that cause Hhcy, can be responsible for long term cardiovascular complications or short-term reproductive outcomes in women with insulin-resistant PCOS [7,9-11]. Preliminary investigations done in PCOS women who were involved in various observational and randomized controlled trials demonstrated high serum homocysteine levels in most of the studies along with other cardiovascular disease serum biomarkers such as high sensitivity C-reactive protein, plasma soluble CD40 ligand (sCD40L), and asymmetric dimethylarginine (ADMA) [12,13]. As discussed above, homocysteine being an independent risk factor for cardiovascular disease and PCOS having long term cardiovascular complications, it is vital to know the association of elevated homocysteine levels in PCOS management.

In this literature review, we aimed to comprehensively discuss the overall association of Hhcy in managing PCOS patients. We discussed the effects of Hhcy on reproductive and cardiovascular components of PCOS, effects of the current treatment of PCOS on Hhcy, and suggested management of Hhcy in PCOS patients. For this, we collected relevant data from the PubMed database using the keywords homocysteine levels, PCOS. We manually screened and included relevant published articles from the PubMed database after using the MeSH strategy, regular keywords search, and combined keyword search (PCOS and Homocysteine levels) and avoided duplication of data.

## Review

Homocysteine levels and insulin resistance in PCOS

Insulin resistance (IR) is the central pathogenetic feature in PCOS and is present in up to 70% of women with PCOS [4,14]. In several clinical situations, plasma insulin levels and increased Hcy levels are positively correlated [11]. It has been implicated that plasma insulin levels influence Hcy levels and its metabolism through effects on glomerular filtration or by inhibition of hepatic cystathione β synthase activity [11,14]. Previous studies have evaluated the relationship between insulin resistance and Hcy levels in PCOS patients. In one such study, Schachter et al. found that Hcy levels were significantly elevated in IR PCOS patients (12.4 ± 8.4 µmol/l) compared with non-IR PCOS patients (9.6 ± 4.4 µmol/l), independent of the significant elevation of Hcy found in all infertile PCOS patients (11.5 ± 7.4 µmol/l) (p<0.001) [11]. A statistically significant correlation was identified between serum Hcy and insulin levels irrespective of BMI at the end of the study [11]. A similar study was done a decade later by Sachan Rekha et al. in infertile women with PCOS and without PCOS(controls), which showed statistically significant elevated levels of Hcy in PCOS and IR PCOS women when compared to controls (p<0.001) [14]. This study also concluded obesity is not an independent risk factor for elevated Hcy levels in PCOS women [14]. Bayraktar F et al. investigated a correlation between Hcy levels, IR, and androgen levels in women with PCOS and non-classic congenital adrenal hyperplasia (CAH) caused by 21-hydroxylase deficiency, as high androgen levels are a common characteristic for both disorders [15]. The study concluded the reason for Hhcy to be related to IR but not androgen levels [15]. A cross-sectional study done by Zhang et al. on 196 Chinese PCOS patients demonstrated that high levels of CD14++CD16+ inflammatory monocytes were associated with Hhcy and IR in PCOS patients [16]. Karadeniz et al. performed a genetic study on ABCA1 gene polymorphism in PCOS patients [17]. Compared with heterozygous and wild genotypes, this study discovered significantly higher levels of fasting insulin and Hcy levels in PCOS patients with the ABCA G1051A mutant genotype [17]. Also, a higher percentage of AA genotype and A allele of ABCA G2706A was found in PCOS patients when compared to controls [17].

In contrast, a study was done by Kilic-Okman et al. on 29 patients and 25 healthy subjects concluded that age, BMI, and IR were not related to levels of Hcy in PCOS patients [18]. They suggested serum Hcy increase independently in PCOS patients [18]. Overall, many studies proposed a significant association between Hcy levels and IR by observing at the cellular level, genetic level, and various other variables like androgen levels and BMI. However, the Kilic-Okman et al. study suggested no relation between Hcy and IR, which may be due to the study's small sample size. Furthermore, although a statistical significance was demonstrated in the studies by Schachter et al. and Sachan Rekha et al., the clinical relevance of these studies remains unclear as the total number of patients involved was low. Because of the limited sample sizes of the above studies and different criteria used to identify and include subjects for the studies, the results cannot be generalized in the PCOS population.

Homocysteine levels and cardiovascular health in PCOS

Hcy, with its primary atherogenic and prothrombotic properties, has a vital role in cardiovascular morbidity and mortality [3]. It exerts its effects through leucocyte recruitment, smooth cell proliferation, enhanced collagen production, foam cell formation, disrupting blood clotting mechanics, endothelial cell injury, and impairing nitric oxide production [3]. Studies have been done to evaluate the homocysteine levels in PCOS patients in the context of cardiovascular health. Yarali et al. performed a prospective case-control study on 30 PCOS patients and 30 healthy controls [19]. This study assessed systolic and diastolic function parameters along with Hcy levels and insulin sensitivity in both study groups, which yielded a trend of non-restrictive type diastolic dysfunction apart from elevated Hcy levels in PCOS women [19]. It concluded that cardiovascular disease risk in PCOS patients might be contributed by diastolic dysfunction discovered in this study, along with increased levels of Hcy in PCOS [19]. A study done by Battaglia C et al. to evaluate subclinical vascular risk in PCOS patients concluded that increased vascular risk is observed in PCOS [20]. It was done in 28 PCOS patients, 17 eumenorrheic polycystic ovaries (PCO) women, and 15 healthy controls, and demonstrated a significantly elevated uterine pulsatility index after doppler analysis apart from elevated Hcy and decreased plasma nitrate levels in PCOS patients [20]. Measurement of plasma soluble CD40 ligand(sCD40L) levels and Hcy levels in 31 patients with PCOS and 31 non-PCOS patients was done by Oktem et al. [13]. CD40 ligand, which belongs to the tumor necrosis family upon engagement with the CD40 receptor, promotes atherosclerosis, which is the leading cause of cardiovascular disease [13]. Results showed significantly higher levels of sCD40L and Hcy levels in PCOS patients when compared to controls(p<0.05) [13]. Mohamadin et al. studied plasma markers of cardiovascular disease in 50 Saudi women with PCOS and 40 controls without PCOS, emphasizing asymmetric dimethylarginine (ADMA) and Hcy levels [21]. A significantly elevated level of ADMA, which is a sensitive cardiovascular risk marker, as well as elevated Hcy level and lipid profile, were observed in the study (p<0.001) when compared with controls [21]. The study also concluded that clinicians should follow up on these markers to manage PCOS in reducing cardiovascular risk [21]. A study performed by Kaya et al. in 68 PCOS women and 68 healthy controls demonstrated an abnormal heart rate recovery (HRR) in PCOS patients who have elevated levels of Hcy and C reactive protein [22]. A case-control study done by Lin et al. on 339 women, of which 84 women have Hhcy and 255 women having normal Hcy, identified a correlation between serum Hcy, serum total testosterone levels, and diastolic blood pressure [23]. Hhcy and hyperandrogenemia were shown to have a significant association after logistic regression analysis was performed (OR: 2.24, 95% CI: 1.26-4.01) [23]. The association between Hhcy and biochemical hyperandrogenism may contribute to cardiovascular risk in women with PCOS [23].

Yarali et al. and Battaglia et al. identified diastolic dysfunction and vascular risk in PCOS women with Hhcy, respectively [19,20]. Still, because of the limited sample size, it is unclear about their clinical significance. A statistically significant elevation of cardiovascular risk markers like sCD40L, ADMA along with Hcy levels in the studies by Oktem et al. and Mohamadin et al. raises a question of whether to involve those markers in the routine clinical screening of PCOS to evaluate cardiovascular risk [13,21]. However, we suggest larger scale observational studies because of the large prevalence of PCOS in reproductive age women to understand the clinical significance of these markers in the routine management of PCOS. Abnormal HRR observed in the study by Kaya et al. is directly related to increased Hcy levels (p <0.001; 95% CI: 0.026-0.081) [22]. Furthermore, a statistical significance was identified between the Hhcy and hyperandrogenism association, which further increase concerns in the management of PCOS women's cardiovascular health [23].

Homocysteine levels and reproductive health in PCOS

Hhcy in PCOS women is implicated in increased pregnancy loss and reduced ovulation [24]. Hhcy, a risk factor for arterial and venous thrombosis, poses a threat to women with habitual abortion [25]. In PCOS patients undergoing assisted reproduction, the quality of oocytes, fertilization rate, and the quality of embryos are evaluated using concentrations of Hcy levels in follicular fluid [26]. Berker et al. did a study on 52 PCOS patients undergoing assisted reproduction [26]. A total of 94 follicular samples were analyzed, and after multiple linear regression analyses, follicular fluid Hcy level was shown to be an independent predictor of oocyte quality (p < 0.001, 95% CI: −0.25 to 0.09) and embryo quality (p < 0.001, 95% CI: −0.49 to 0.311) [26]. Also, follicular fluid Hcy is negatively correlated with fertilization rate (r=-0.85), follicular fluid vitamin B12 (r = -0.44) and positively correlated with malonyl dialdehyde (MDA) (r= 0.51) [26]. Chakraborty et al. performed a retrospective study on 126 PCOS women with recurrent pregnancy loss (RPL) and 117 non-PCOS women with matched age range as controls to evaluate Hhcy and IR's role in PCOS women with RPL [27]. A significantly higher rate of miscarriage was observed in PCOS women with Hhcy (70.63%) when compared to PCOS women with normal Hcy levels (29.36%) (p<0.008) [27]. A prospective observational study performed by Chakraborty et al. in 336 women with RPL evaluated the difference between aspirin's efficacy versus combined aspirin-low molecular weight heparin (LMWH) in preventing RPL in Hhcy women [25]. The sole outcome measure was pregnancy salvage, which was 43.15% represented by women with normal Hcy, and much lower salvage rate in Hhcy women after aspirin therapy and 66.84% after combined aspirin-LMWH therapy [25]. After logistic regression analysis, it was determined that Hhcy was a significant predictor for pregnancy salvage rates in PCOS women [25]. The study concluded that in Hhcy women, combined aspirin-LMWH therapy conferred added benefit in preventing RPL [25]. A study was done in JIPMER, Puducherry by Chitra et al. on 50 PCOS patients admitted for laparoscopic ovarian drilling (LOD) evaluated LOD's effects on serum Hcy levels and clinical pregnancy outcomes in PCOS women [28]. This study demonstrated a reduction in Hcy levels (p < 0.001) with a significant reduction in conceived groups (p < 0.001) when compared to non-conceived groups, improved clinical pregnancy(21/50), and ovulation rate (38/50) outcomes after LOD [28]. 

Based on Berker et al. study, increased follicular Hcy levels are a significant predictor of the oocyte, embryo quality, and fertilization rate in PCOS women undergoing assisted reproduction [26]. Two different studies done by Chakraborty P et al. proposed that higher miscarriage rates are seen in PCOS women with Hhcy, and combined aspirin-LMWH therapy benefitted Hhcy PCOS women in the prevention of RPL [25,27]. Therefore, we believe Hcy levels in serum and follicular fluid play a significant role in managing PCOS patients' reproductive health. Chitra et al. performed LOD, which yielded a considerable reduction in Hcy levels and improved clinical outcomes in PCOS women [28]. Because of this therapy's invasive nature and limited sample size in the study, it is not clear about its application on a larger scale. Based on the above studies' data, we believe investigating Hcy levels in managing PCOS patients' reproductive health plays a vital role in improving overall clinical outcomes. 

Effects of PCOS treatment on homocysteine levels

Some studies were done to evaluate the effects of PCOS treatment on serum Hcy levels. One such study done by Vrbíková et al. examined serum Hcy levels in PCOS patients treated with metformin [29]. Results showed significant elevation in Hcy levels (10.1 +/- 2.6 to 13.4 +/- 5.1 micromol/l, p < 0.05) after metformin treatment in nine PCOS women [29]. A randomized study was done by Kilicdag et al. in 30 PCOS patients randomized into two groups receiving two different treatments, metformin or rosiglitazone, and evaluated their effects on serum Hcy levels [30]. It demonstrated a significantly increased level of Hcy in both treatment groups (metformin- p = 0.002 and rosiglitazone- p = 0.01) [30]. The study concluded that treatment with insulin sensitizers could contribute to elevated Hcy levels in women with PCOS [30]. Palomba et al. performed a non-randomized double-blind placebo-controlled clinical study on 50 PCOS patients divided into two treatment groups with 25 PCOS patients treated with metformin +folic acid (experimental group) and other 25 patients on metformin+placebo(control group) for six months [10]. Significantly higher Hcy levels were observed in the control group than the experimental group (p<0.05) [10]. The researchers concluded that the addition of folic acid to metformin provided extra benefit to vascular endothelium [10]. An interventional study was performed by Esmaeilzadeh et al. on 18 PCOS patients treated with metformin for six months [31]. Assessment of serum Hcy levels, vitamin B12, folic acid levels after treatment with metformin demonstrated a significant decrease in vitamin B 12 levels (p<0.002), and an increase in Hcy levels with statistically significant elevation observed in women with BMI >25 kg/m2 (p=0.01) when compared to before treatment [31]. Harmanci et al. determined that estradiol/drospirenone (EE-DRSP) plus spironolactone therapy for six months in PCOS women improve androgen excess in lean PCOS patients but increase the serum Hcy levels (13.1 ± 5.2 vs. 17.6 ± 5.3 μm, p < 0.05) [32].

In contrast to the above studies, few studies suggested that Hcy levels are unaffected or decreased by metformin treatment or oral contraceptive treatment in PCOS patients. A study done by Riahinejad et al. demonstrated a significant reduction in Hcy levels (p<0.05) after treating with metformin for three months in 33 random PCOS patients [33]. Two prospective randomized placebo-controlled studies done by Carlsen et al. on 63 infertile PCOS women and 38 pregnant PCOS patients demonstrated no effect of metformin on Hcy levels both in non-pregnant and pregnant PCOS women [34]. Yilmaz et al. proposed no change in Hcy level after treating with metformin or rosiglitazone for three months in a sample size of 50 lean PCOS women divided into two groups for study along with 35 healthy subjects as controls [35]. Based on all the above data, the effects of PCOS treatment on serum Hcy levels appear controversial. Even though the above studies resulted in statistical significance regarding Hcy levels after specific treatment, it is unclear whether to evaluate serum Hcy levels in the management of all PCOS women undergoing treatment due to varying inclusion criteria of subjects used for the studies and limited study sample sizes. However, we believe it is essential to follow up on Hcy levels in high-risk PCOS patients undergoing treatment and report any observed abnormal differences in Hcy levels during or after therapy to build up data for future studies.

Key studies that focused on the treatment of hyperhomocysteinemia in PCOS women

Studies listed in Table 1 provide an overview of various treatment regimens investigated in the past in PCOS women, that significantly impact levels of Hcy after therapy for a specified time. This data provides clinicians with valuable information in treating Hhcy in PCOS women associated with insulin resistance, cardiovascular, metabolic, and reproductive health risk.

**Table 1:** List of studies focused on the treatment of hyperhomocysteinemia in PCOS women MI: myoinositol; Hcy: homocysteine; Hhcy: hyperhomocysteinemia; IR: insulin resistance; NIR: noninsulin resistance; EE+CA: ethinyl estradiol + cyproterone acetate; hsCRP: high sensitivity C- reactive protein; PCOS, polycystic ovarian syndrome

No.	Author name	Year of publication	Total sample size	Objective and type of study	Results	Conclusion of the study
1.	Randeva et al. [36]	2002	21 young obese PCOS women (12- exercisers, 9-nonexercisers)	Effect of six-month exercise program on total plasma Hcy levels in young overweight or obese PCOS women	They observed a statistically significant decrease in plasma Hcy levels(p<0.001) in the exercise group compared to baseline.	The study concluded that regular exercise lowers plasma Hcy levels significantly in young obese or overweight PCOS women
2.	Kilicdag et al. [37]	2005	(60 PCOS women) Group 1-20 Group 2-20 Group 3-20	A randomized trial done to assess the effects of administration of B group Vitamins and folic acid on serum Hcy levels in metformin taking PCOS women.	21.17% and 8.33% reduction in Hcy levels were observed in women supplemented with B group vitamins or folic acid+ metformin, respectively. A 26.5% increase in Hcy level was observed in women taking only metformin in three months.	B group vitamins or folic acid supplementation counteracted the metformin's Hcy elevation.
3.	Gul et al. [38]	2008	30 PCOS women	A prospective study evaluated the effect of oral contraceptive ethinyl estradiol-cyproterone acetate (EE-CA) on serum Hcy level in PCOS women.	EE-CA therapy for three months showed a statistically significant reduction in Hcy levels(p=0.01).	The study proposed that further studies are required to know the clinical benefit of this (EE-CA) therapy even though the decrease in Hcy level was statistically significant.
4.	Kazerooni et al. [39]	2008	70 Hhcy PCOS women (32-IR, 38-NIR)	A prospective clinical trial was done to investigate folic acid's effect in Hhcy PCOS women with IR and NIR after three-month therapy.	After treatment, serum Hcy levels were significantly decreased in both groups. (p<0.001-IR, p<0.001- NIR)	It was concluded that after folic acid therapy for three months, serum Hcy levels in Hhcy PCOS women significantly reduced with more reduction in NIR PCOS women.
5.	Kaya et al. [40]	2009	52 PCOS women Group 1- 26 Group 2- 26 52 controls (age & BMI matched)	A prospective randomized study performed to test the hypothesis Hhcy in PCOS women improved with statins.	Twelve weeks of treatment with atorvastatin (group 1) & simvastatin (group 2) significantly decreased Hcy levels(p<0.01) in PCOS women with more decrease observed in the atorvastatin group.	The study proposed that statin therapy helps in the reduction of serum Hcy levels in PCOS women.
6.	Makedos et al. [41]	2010	83 PCOS women (divided into five groups for five different regimes)	A prospective matched study done to assess the effects of five different hormonal regimes on serum Hcy and serum hsCRP levels in PCOS women.	All five treatment regimens 1. Conjugated estrogens+ cyproterone acetate, 2. 17β-estradiol + cyproterone acetate, 3. Ethinyl estradiol+ cyproterone acetate (high dose), 4. Ethinyl estradiol+ cyproterone acetate (low dose), 5. Ethinyl estradiol+ desogetrel, demonstrated significant reduction of Hcy levels(p< 0.001) when measured after four, seven, 12 months	Even though all five regimens favored the reduction of Hcy levels, it concluded that 17β-estradiol + cyproterone acetate regime had the most favorable effect in PCOS women in terms of both Hcy and hsCRP levels reduction.
7.	Bahmani et al. [42]	2014	69 PCOS women (18-40 years age) (Divided into three equal groups) Group 1- folate 1 mg/day Group 2- folate 5 mg/day, Group 3-placebo	A randomized, double-blinded placebo-controlled clinical trial conducted to assess the effects of folate therapy for eight weeks on inflammatory factors and biomarkers of oxidative stress in PCOS women.	The study resulted in a statistically significant decrease in Hcy levels in women treated with 5mg/day folate (p<0.05) compared with other treatment groups.	Therefore, 5mg/day folate Supplementation had favorable effects on inflammatory and oxidative stress biomarkers in PCOS women.
8.	Salehpour et al. [43]	2016	50 PCOS women	This pilot study was done to evaluate the effects of myoinositol (MI) on the cardiovascular and metabolic profile of PCOS women over 30 years of age.	After three months of therapy with MI 2 g + 200 μg of folic acid, a statistically significant reduction of serum Hcy levels(p<0.008) was observed along with significant improvement in other metabolic and cardiovascular parameters of PCOS women.	Even though a statistically significant reduction in Hcy levels and improved other parameters were observed after treatment with MI+ folic acid, they suggested further studies on a larger population to apply therapy clinically.
9.	Stracquadanio et al. [44]	2018	100 PCOS women	The study aimed to compare clinical, metabolic, endocrine parameters in two treatment groups of PCOS women after six months of continuous therapy. Group A- MI+ gymnemic acid + L methyl folate Group B- MI + folic acid only.	Metabolic assessment in the results showed a significant decrease in Hcy levels and total cholesterol levels in group A.	Study concluded that the combination therapy (MI+ gymnemic acid + L methyl folate) could be a good option for obese PCOS women with marked IR and Hhcy.

A statistically significant reduction in Hcy levels was observed in PCOS women doing regular exercise or therapy with B group vitamins, folic acid (5 mg/day), or statins therapy (especially atorvastatin) or EE+CA (ethinyl estradiol + cyproterone acetate) therapy or MI (myoinositol) + folic acid therapy for a specified amount of time. A recent study done in 2018 by Stracquadanio et al. investigated a combination therapy of MI + gymnemic acid + L methyl folate in PCOS women with IR and Hhcy [44]. It is postulated that Gymnemic acid, with its anti-diabetic properties, anti-inflammatory, and delay glucose absorption from blood, has a synergistic action to myoinositol's insulin-mimetic properties [44]. Also, in contrast to folic acid, L methyl folate has higher bioavailability, absorption rate, and no drug/food interactions [44]. As the study was done in 100 PCOS women, given the higher prevalence rates of PCOS, we suggest a randomized clinical trial on a larger scale to know the clinical implication of this combination therapy.

## Conclusions

Homocysteine being an independent risk factor for atherogenic and thrombotic components of various systems and many past studies identifying an association between PCOS and Hcy, we aimed through this article to review the overall importance of Hcy levels in association with various components of PCOS. IR PCOS women demonstrated higher Hcy levels when compared to NIR PCOS women in multiple studies. We believe excess serum Hcy levels add extra risk to PCOS women's cardiovascular health as studies identified diastolic dysfunction, increased vascular risk, Hcy levels associated with excess androgen, and elevated Hcy levels along with other cardiovascular risk markers in PCOS women. Use of aspirin-LMWH combination improved RPL in Hhcy PCOS women, and levels of Hcy are used as a predictor for oocyte, embryo quality, and fertilization rates in assisted reproduction of PCOS women. It is possible that commonly used PCOS treatment regimens like metformin can affect serum Hcy levels. These findings concern the role of Hcy levels in managing PCOS women and raise a question if the broader application of investigating Hcy levels and treating the Hhcy in PCOS women favors the course of PCOS management in the long run. We recommend an observational study on a larger scale to know the prevalence of elevated Hcy levels in a well-defined larger PCOS population in order to include it in routine clinical practice.
